# Entomological assessment of dengue virus transmission risk in three urban areas of Kenya

**DOI:** 10.1371/journal.pntd.0007686

**Published:** 2019-08-23

**Authors:** Sheila B. Agha, David P. Tchouassi, Michael J. Turell, Armanda D. S. Bastos, Rosemary Sang

**Affiliations:** 1 International Centre of Insect Physiology and Ecology, Nairobi, Kenya; 2 Department of Zoology and Entomology, University of Pretoria, Hatfield, South Africa; 3 VectorID LLC, Frederick, Maryland, United States of America; 4 Center for Virus Research, Kenya Medical Research Institute, Nairobi, Kenya; University of Washington, UNITED STATES

## Abstract

Urbanization is one of the major drivers of dengue epidemics globally. In Kenya, an intriguing pattern of urban dengue virus epidemics has been documented in which recurrent epidemics are reported from the coastal city of Mombasa, whereas no outbreaks occur in the two major inland cities of Kisumu and Nairobi. In an attempt to understand the entomological risk factors underlying the observed urban dengue epidemic pattern in Kenya, we evaluated vector density, human feeding patterns, vector genetics, and prevailing environmental temperature to establish how these may interact with one another to shape the disease transmission pattern. We determined that (i) Nairobi and Kisumu had lower vector density and human blood indices, respectively, than Mombasa, (ii) vector competence for dengue-2 virus was comparable among *Ae*. *aegypti* populations from the three cities, with no discernible association between susceptibility and vector cytochrome c oxidase subunit 1 gene variation, and (iii) vector competence was temperature-dependent. Our study suggests that lower temperature and *Ae*. *aegypti* vector density in Nairobi may be responsible for the absence of dengue outbreaks in the capital city, whereas differences in feeding behavior, but not vector competence, temperature, or vector density, contribute in part to the observed recurrent dengue epidemics in coastal Mombasa compared to Kisumu.

## Author summary

Dengue is a viral disease of global public health significance owing to rapid spread and increasing disease burden. Urbanization is an important risk factor for dengue emergence. In Kenya, repeated outbreaks of the disease have occurred in the urban areas of Mombasa but not in Nairobi and Kisumu, despite the presence of susceptible human hosts and the primary vector, *Aedes aegypti* throughout these areas. We set out to determine whether this trend is related to variations in biological parameters of the vector, *Ae*. *aegypti* between these areas. Our findings show that (i) *Ae*. *aegypti* had lower density and human blood feeding ability in Nairobi and Kisumu, respectively, compared to Mombasa, (ii) the ability of *Ae*. *aegypti* populations from the three cities to transmit dengue-2 virus was comparable with no observed association between susceptibility and vector genetic variation, and (iii) vector competence was temperature-dependent. Based on this, it appears that higher temperature and *Ae*. *aegypti* vector density explains the higher risk of dengue virus transmission in Mombasa, compared to Nairobi, whereas differences in feeding behavior of *Ae*. *aegypti* may be responsible for the lower risk at Kisumu.

## Introduction

Dengue virus (DENV) is a global public health threat with epidemics mostly reported in urban and semi-urban areas [1,2]. Dengue virus consists of four related serotypes (DENV-1-4), belonging to the genus *Flavivirus* (Family: *Flaviviridae*) [3]. The most recent epidemics in Africa have predominantly been reported in East African countries, with DENV-2 responsible for the highest number of epidemics [4,5]. Dengue epidemics have been linked to urbanization, globalization, climate change, and the broad distributional range of the primary vector, *Aedes aegypti* [6–9].

There are a number of factors such as temperature, vector bionomics (survival, density, feeding frequency/behavior), extrinsic incubation period (EIP), and vector competence that can affect DENV transmission [10–17]. Whilst determination of individual factors is valuable, they are rarely studied in parallel, yet their combined effects may be critical to fully understanding the complex interrelationships influencing DENV transmission risk. Studies investigating the various dengue risk factors in parallel are lacking in many endemic areas, including Kenya.

In the last decade, dengue has re-emerged as one on the most important vector-borne diseases in Kenya, with recurrent urban outbreaks occurring in coastal areas, particularly in and around the city of Mombasa [18–20]. In contrast, no outbreaks have been reported in the other major cities of Kisumu and Nairobi, in spite of population movement between cities. Previous entomological studies in Kenya have examined risk of DENV transmission by studying vector density/abundance and vector competence data separately [10–12,20]. However, the observed differential dengue outbreak pattern in these urban areas remains unexplained.

In this study, different risk parameters related to the DENV vector in the three major cities of Kenya; Mombasa, Kisumu, and Nairobi were studied in parallel to gain a better understanding on their potential influence on the observed differential outbreak patterns. We hypothesized that 1) the *Ae*. *aegypti* vector density differs between these three cities, 2) the ability of *Ae*. *aegypti* to transmit DENV-2 (vector competence) differs between populations from these cities and may be influenced by temperature, 3) the *Ae*. *aegypti* populations in the three cities differed in their anthropophilic behavior, and 4) there is an underlying population genetic component to DENV-2 susceptibility of *Ae*. *aegypti* in each city. An improved understanding of the factors responsible for differences in dengue outbreak risk is key to informing targeted interventions and preventing future outbreaks in the urban areas of Kenya.

## Materials and methods

### Ethics statement

Scientific and ethical approval was obtained from Kenya Medical Research Institute Scientific and Ethics Review Unit (KEMRI-SERU) (Project Number SERU 2787). We sought permission from household heads through oral informed consent to allow their residences to be surveyed for mosquitoes. The animal use component was reviewed and approved by the KEMRI Animal care and use committee (KEMRI ACUC) (approval number KEMRI/ACUC/ 03.03.14) through the KEMRI-SERU review process. The KEMRI ACUC ensures adherence to national guidelines on the care and use of animals in research and education in Kenya enforced by National Commission for Science, Technology and Innovation (NACOSTI). The Institute has a foreign assurance identification number F16-00211 (A5879-01) from the Office of Laboratory Animal Welfare under the Public Health Service and commits to the International Guiding Principles for Biomedical Research Involving Animals.

### Study area

This study was carried out in three major cities of Kenya; Mombasa (dengue endemic, average monthly temperature 27–31°C), Kisumu, and Nairobi (no dengue outbreak reports, average monthly temperatures of 28–30°C and 22–28°C, respectively). Mombasa (4°03'S 39°40'E, population 1.2 million people) is Kenya’s second largest city and is located on the coast. Apart from being a major tourist site, Mombasa is also an important port city. Inland Nairobi (01°17'S36°48'E, population 3.1 million people) is the capital city of Kenya. Kisumu (0°03′S34°45′E, population >400,000) is located on the shores of Lake Victoria and is the third largest city in Kenya. All three cities are characterized by the presence of a national/international airport and thus serve as local, regional, and international transport hubs. These cities serve as main gateways to East Africa, and due to the ease of interconnectivity, we would expect periodic generation of dengue epidemics in all three cities resulting from either importation of infectious cases or infected vectors into these cities from within/outside the country [8]. This, however, has not been the case in Kenya, and epidemics (of DENV-1, DENV-2, and DENV-3) remain primarily limited to the city of Mombasa, with DENV-1 and DENV-2 responsible for the highest number of cases. All three cities experience three seasons; the long-rain (April-June), the short-rain (October-December), and the dry (January-March and July-September) seasons. Outbreaks that have occurred in Mombasa have mostly been reported during the long-rain season [20].

**Fig 1:**
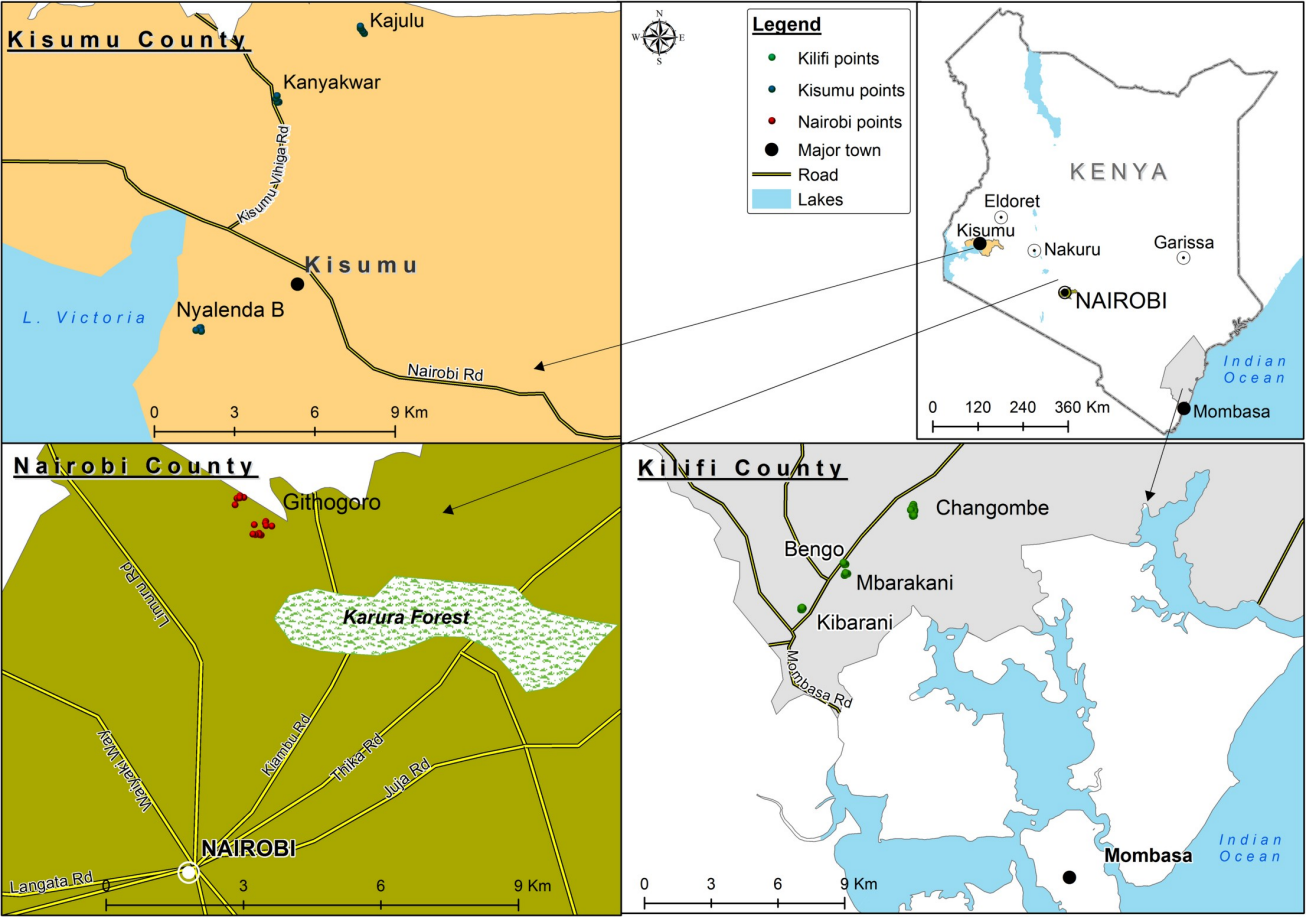
Map showing the study sites within the urban areas of Kisumu, Mombasa, and Nairobi in Kenya [10].

In Mombasa, we specifically selected sites from around the city (Rabai-Kilifi) based on previous history of DENV-2 circulation [19]. In Kisumu and Nairobi, selection of sites was partly informed by logistical constraints, such as ease of access to homes. The study sites were Kanyakwar, Kajulu, and Nyalenda B in Kisumu and Githogoro in Nairobi (Fig 1).

### *Aedes aegypti* density

Host seeking *Ae*. *aegypti* mosquitoes were collected from Mombasa, Kisumu, and Nairobi during the long-rain (April-June), short-rain (October-December), and dry (January-March and July-September) seasons, during 2014–2016. The seasons were defined by the average amount of rainfall two weeks prior to mosquito sampling. The data was obtained from the Kenya Meteorological Department and the values were 12.4, 10.8 and 8.3 mm during the long-rains, 5.5, 4.0 and 7.3 mm during the short-rains and 0, 0.3 and 0 mm during the dry season in Mombasa, Kisumu and Nairobi, respectively.

Briefly, BG-Sentinel traps (BioQuip Products, Rancho Dominiguez, CA, USA) were baited with carbon dioxide in the form of dry ice. In each city, 12 traps were set up in vegetation close to human habitation at our selected sites at 7 am and retrieved at 6 pm on the same day [11]. This was done for five consecutive days in each season in each city, translating to 180 traps per city (60 traps per season). Alongside the BG trapping, *Ae*. *aegypti* mosquitoes were collected indoors (sitting room, bedroom, and kitchen) using a battery powered Prokopack aspirator (BioQuip Products, Rancho Dominguez, CA, USA) from 150 houses per city (50 per season). Aspiration was done between 11am and 3pm, and lasted about 20 minutes per house.

Mosquitoes were morphologically identified using taxonomic keys [21–23] at the International Centre of Insect Physiology and Ecology (*icipe*) as previously described [11]. The number of female *Ae*. *aegypti* mosquitoes collected was recorded and used to estimate the vector density. This was done by dividing the total number of female *Ae*. *aegypti* mosquitoes collected by the number of traps for each city. Similarly, the blood-fed *Ae*. *aegypti* mosquitoes trapped were used to perform a blood meal analysis.

### *Aedes aegypti* blood meal analysis

In addition to mosquito collection conducted using the BG-Sentinel traps, we also attempted to collect blood-fed mosquitoes using a prokopack aspirator indoors, outdoors, and on the nearby vegetation from 150 houses per city (50 per season). As no blood-fed *Ae*. *aegypti* mosquito were collected using the aspirator approach [11], blood meal analyses were performed on wild-caught, blood-fed *Ae*. *aegypti* mosquitoes collected using BG-Sentinel traps, only. The abdomen of individual mosquitoes was cut using a scalpel, sterilized with 70% ethanol between specimens to prevent cross contamination of samples. Genomic DNA was extracted from whole blood contained in individual mosquito abdomens using the DNeasy blood and tissue Kit (Qiagen, GmbH-Hilden, Germany) as per the manufacturer’s recommendation. The extracted DNA was used as a template for amplification of a 500 bp fragment of the mitochondrial 12S rRNA gene (S1 Protocol), a target used for mammalian blood meal identification [24]. Amplicons were individually purified using ExoSap PCR purification kit (USB Corp., Cleveland, OH). Unidirectional sequencing (forward strand) was outsourced to a commercial company (Inqaba biotec, Pretoria, South Africa). Sequences were evaluated through BLAST nucleotide searches against the Genbank database (www.ncbi.nlm.nih.gov/blast) in order to identify the closest sequence matches (threshold > 98%) and infer the species identity of the blood meal.

To increase the sample size of blood fed mosquitoes, an additional sampling was done using the BG-Sentinel traps for 7 consecutive days (12 traps per day) during the long-rain season (April-June) in 2018, for the three cities. These mosquitoes, which were used for host blood meal determination alone, were processed for blood meal identification in the same manner as the blood-fed mosquitoes collected during 2014–2016.

### Vector competence of *Aedes aegypti* mosquitoes for dengue virus

#### Mosquito colonies

We tested *Ae*. *aegypti* mosquitoes from Mombasa, Kisumu, and Nairobi for their susceptibility to DENV-2. These mosquitoes were collected as immatures from water holding containers in and around houses between October-December 2016. Immature mosquitoes were reared to F_0_ adults in a BSL-2 insectary at *icipe*, maintained at 28°C and a 12:12 (L:D) photoperiod [25]. After the identity of the adult mosquitoes was confirmed as *Ae*. *aegypti*, they were blood-fed on laboratory mice (Kenya Medical Research Institute, Animal house) to provide eggs. The eggs were hatched in dechlorinated tap water, and the emerging larvae were fed once a day on Tetramin fish food (Tetra, USA). To obtain F_2_ mosquitoes, the same procedure was repeated for the emerging F_1_ adults. The F_2_ mosquito generation was used in the vector competence study for all three cities.

#### Dengue virus strain and assay

The DENV-2 strain used in this study (Sample number: 008/01/2012) was isolated during the 2012 outbreak in Mandera, Kenya. The virus had been passaged twice on C6/36 cells and twice on Vero E6 cells grown in cell culture media consisting of Minimum Essential Media-MEM (Sigma-Aldrich, St. Louis, MO), with Earle's salts and reduced NaHCO3, supplemented with 10% heat-inactivated fetal bovine serum (FBS) (Sigma-Aldrich), 2% L-glutamine (Sigma-Aldrich), and 2% antibiotic/antimycotic solution with 10,000 units penicillin, 10 mg streptomycin and 25μg amphotericin B per ml (Sigma- Aldrich) [25]. This virus stock, with a titer of 10^4.3^ plaque-forming units (PFU)/ml, was kept frozen at -80°C. To produce infectious virus for the vector competence study, a vial of this stock virus was used to inoculate freshly grown Vero E6 cells in a T-25 cell culture flask (Corning Incorporated, USA). Virus adsorption was achieved by incubating the cells at 37°C for 1 hour. The cells were overlaid with maintenance media (MEM supplemented with 2% FBS), and incubated at 37°C in a 5% CO_2_ incubator. Cells were observed daily for peak viral levels and harvested by day 7, after observing 80% cythopathic effect (CPE). The DENV-2 media suspension was harvested for direct use, without freezing, in the mosquito infectious blood meal trials.

#### *Aedes aegypti* infection assay

Pre-starved mosquitoes (24 hours), aged 4–9 days, from all three cities were exposed to 2 ml (per well of the hemotek membrane feeder) of an infectious blood meal consisting 1:2 parts of freshly harvested DENV-2 media suspension and defibrinated sheep blood (Central Veterinary Laboratories Kabete, Kenya). The artificial feeder (hometek membrane feeder) was covered with mouse skin (Kenya Medical Research Institute, Animal house). After 1 hour of feeding, fully blood-fed mosquitoes, originating from each of the three cities under study, were removed from the feeding cage, and divided into three new cages, each of which was incubated at either 22°C, 28°C, or 31°C. These three temperature treatments are representative of the minimum/maximum average monthly temperature of each source city; 22°C—minimum temperature in Nairobi, 28°C—maximum temperature in Nairobi, and minimum temperature in Kisumu and Mombasa, 31°C—maximum temperature in Kisumu and Mombasa. A proportion of the mosquitoes originating from the different cities were sampled on days 7, 14, and 21 (for each of the incubation temperatures) and tested for infection, dissemination, and transmission (by the capillary tube method) [25] of DENV-2 (S2 Protocol). Three replicates of the experiment were performed. Pre- and post-feeding blood/virus mixtures were collected to quantify the virus to which the mosquitoes were exposed (S2 Protocol).

#### Cytochrome c oxidase subunit 1 (*COI*) variation in *Aedes aegypti* populations

To determine possible genetic differences between DENV susceptible and non-susceptible *Ae*. *aegypti* mosquito populations from the three cities, genomic DNA was extracted from the body homogenate of *Ae*. *aegypti* mosquitoes (susceptible and non-susceptible to DENV-2), using DNeasy blood and tissue Kit (Qiagen, GmbH-Hilden, Germany). From the vector competence experiment, mosquitoes with DENV-2 positive bodies were considered susceptible to DENV-2 infection while those with a negative body were considered non-susceptible. The 860 bp barcode region of the cytochrome c oxidase subunit 1 (*COI*) gene was amplified using published primers [26] and outsourced to Macrogen (Seoul, Republic of Korea) for Sanger sequencing (unidirectional sequencing–forward strand). Sequences were, viewed and edited in Chromas, prior to phylogenetic analysis using MEGA v 5 software [27]. Homologous sequences in the Genbank database were identified through BlastN searches and aligned using ClustalW in MEGA v 5 to reference *CO1* gene sequences for domestic *Ae*. *aegypti* (Genbank No. AF390098 and MF194022) and *Ae*. *aegypti formosus* (Genbank Accession No. AY056597). The GTR+G model of sequence evolution was used to infer a Maximum Likelihood (ML) tree in MEGA v 5 and guided selection of priors for Bayesian inference (BI) with MrBayes 3.2 [28]. Nodal support was assessed through 5,000 bootstrap replications for ML and from Bayesian posterior probabilities obtained from two independent runs of 20 million generations each, with burn-in set to 25%, for the BI analyses. The haplotypes generated in this study were deposited in GenBank under accession numbers MH410177 –MH410212.

### Statistical analyses

*Aedes aegypti* density (total number of female *Ae*. *aegypti* per trap) was estimated and the difference between the cities compared using a t-test.

Recovery of virus from the mosquito’s body and not legs confirmed that the mosquito had a non-disseminated infection limited to the midgut. Recovery of virus in the body and legs was considered as a disseminated infection [25]. Mosquitoes with positive saliva were considered competent in transmitting DENV-2. The overall dissemination and transmission rates at each temperature were compared for the different cities using Fisher’s Exact test. Human blood feeding rates were compared between the cities using Chi-Square test. All analyses were performed in R version 3.3.1 [29] at α = 0.05 level of significance.

## Results

### *Aedes aegypti* density

Based on the total number of female *Ae*. *aegypti* mosquitoes collected using the BG-Sentinel traps from each of the three cities (n = 1,432, n = 1,686, and n = 661 in Mombasa, Kisumu, and Nairobi respectively), the estimated vector density per trap was comparable in Mombasa and Kisumu, 8.0 and 9.4 *Ae*. *aegypti*/trap (T-test, p = 0.186), with each being ~ 2-fold higher than in Nairobi, 3.7 *Ae*. *aegypti*/trap (T-test, p < 0.001) (Table 1). The total number of female *Ae*. *aegypti* collected indoors using the prokopack aspirator was quite low (n = 5, n = 1, and n = 0 for Mombasa, Kisumu, and Nairobi respectively), and these data were not considered in the estimation of vector density.

**Table 1:** Female *Aedes aegypti* mosquitoes collected seasonally using CO_2_-baited BG-Sentinel traps in Mombasa, Kisumu, and Nairobi between October 2014 and June 2016.

City	Long-rain season	Short-rain season	Dry-season	Total	Density (Total no. of female/180 trap)
Mombasa	995	350	87	1432	8.0
Kisumu	1266	194	226	1686	9.4
Nairobi	534	94	33	661	3.7

### *Aedes aegypti* susceptibility to dengue-2 virus

To test if *Ae*. *aeypti* mosquitoes from Mombasa, Kisumu, and Nairobi were able to transmit DENV-2, a total of 505 mosquitoes were exposed to an infectious blood meal with average titers of 10^6.9–7.1^ PFU/ml and evaluated at the minimum/maximum temperatures of the three study cities. Although there was no significant difference in dissemination or transmission rates between mosquitoes from each of the three cities, both dissemination and transmission rates increased with an increase in holding temperature (Table 2). For mosquitoes held at 22, 28, or 31°C, dissemination rates were 2/140 (1%), 25/182 (14%), and 27/183 (15%), respectively. The dissemination rates for mosquitoes held at either 28 or 31°C were significantly higher than the dissemination rate for mosquitoes held at 22°C (Fisher’s exact test, p < 0.0001). For mosquitoes held at 22, 28, or 31°C, transmission rates were 1/140 (1%), 2/179 (1%), and 9/179 (5%), respectively. Similarly, mosquitoes held at 31°C had a significantly higher transmission rate (Fisher’s exact test, p = 0.048) than those held at 22°C, and a higher transmission rate that approached significance (Fisher’s exact test, p = 0.061) when compared to those held at 28°C. Of relevance is that with the exception of two mosquitoes from Mombasa, none of the mosquitoes held at 22°C developed a disseminated infection, and only one of the two (1/140 for all those tested at 22°C) transmitted the virus.

**Table 2:** Vector competence of *Aedes aegypti* originating from three Kenyan cities, exposed to dengue-2 virus and incubated at selected temperatures.

Origin	Infection rate*	Dissem rate^†^	Dissem(I) rate‡	Trans rate§	Trans(I) rate^¶^	Trans(D) rate^#^
**Mosquitoes held at 22°C**						
Mombasa	26 (14/53)	4 (2/53)	14 (2/14)	2 (1/53)	7 (1/14)	50 (1/2)
Kisumu	42 (19/45)	0 (0/45)	0 (0/19)	0 (0/45)	0 (0/19)	n.a.
Nairobi	12 (5/42)	0 (0/42)	0 (0/5)	0 (0/42)	0 (0/5)	n.a.
**Totals**	**27 (38/140)**	**1 (2/140)**	**5 (2/38)**	**1 (1/141)**	**3 (1/38)**	**50 (1/2)**
**Mosquitoes held at 28°C**						
Mombasa	44 (19/43)**	14 (6/43)	32 (6/19)	3 (1/40)	5 (1/19)	17 (1/6)
Kisumu	28 (23/83)	14 (12/83)	52 (12/23)	1 (1/83)	4 (1/23)	8 (1/12)
Nairobi	25 (14/56)	13 (7/56)	50 (7/14)	0 (0/56)	0 (0/14)	0 (0/7)
**Totals**	**31 (56/182)**	**14 (25/182)**	**45 (25/56)**	**1 (2/179)**	**4 (2/56)**	**8 (2/25)**
**Mosquitoes held at 31°C**						
Mombasa	25 (15/61)	10 (6/61)	40 (6/15)	3 (2/61)	13 (2/15)	33 (2/6)
Kisumu	44 (28/63)^††^	17 (11/63)	39 (11/28)	7 (4/61)	14 (4/28)	36 (4/11)
Nairobi	24 (14/59)^††^	19 (11/59)	79 (11/14)	5 (3/57)	21 (3/14)	27 (3/11)
**Totals**	**31 (57/183)**	**15 (28/183)**	**49 (28/57)**	**5 (9/179)**	**16 (9/57)**	**32 (9/28)**

n.a. = not applicable, dissem = dissemination, trans = transmission

*Infection rate: Percent of mosquitoes infected (No. infected/No. tested).

^†^Dissemination rate: Percent of mosquitoes with a disseminated infection (No. disseminated/No. tested).

^‡^Dissemination (I) rate: Percent of infected mosquitoes with a disseminated infection (No. disseminated/No. infected). Measure of midgut escape

barrier.

^§^Transmission rate: Percent of mosquitoes with virus in their saliva (No. transmitting/No. tested).

^¶^Transmission (I) rate: Percent of infected mosquitoes with virus in their saliva (No. transmitting/No. infected).

^#^Transmission (D) rate: Percent of mosquitoes with disseminated infection with virus in their saliva (No. transmitting/No. disseminated).

Measure of salivary gland barrier.

**Includes three mosquitoes not tested for transmission.

^††^Includes two mosquitoes not tested for transmission.

At 22°C, no viral dissemination was observed in any of the populations at 7 days post-exposure. However, by day 14, viral dissemination and transmission were observed exclusively in those mosquitoes originating from Mombasa, but at rates that did not differ significantly from the other two locations. Further, while no virus transmission was observed in the Nairobi mosquito population at 28°C, virus transmission was observed for the Mombasa and Kisumu populations by day 14. At 31°C virus transmission was observed in all three populations by day 7 (S2 Table).

### *Aedes aegypti* host blood feeding pattern

**Fig 2:**
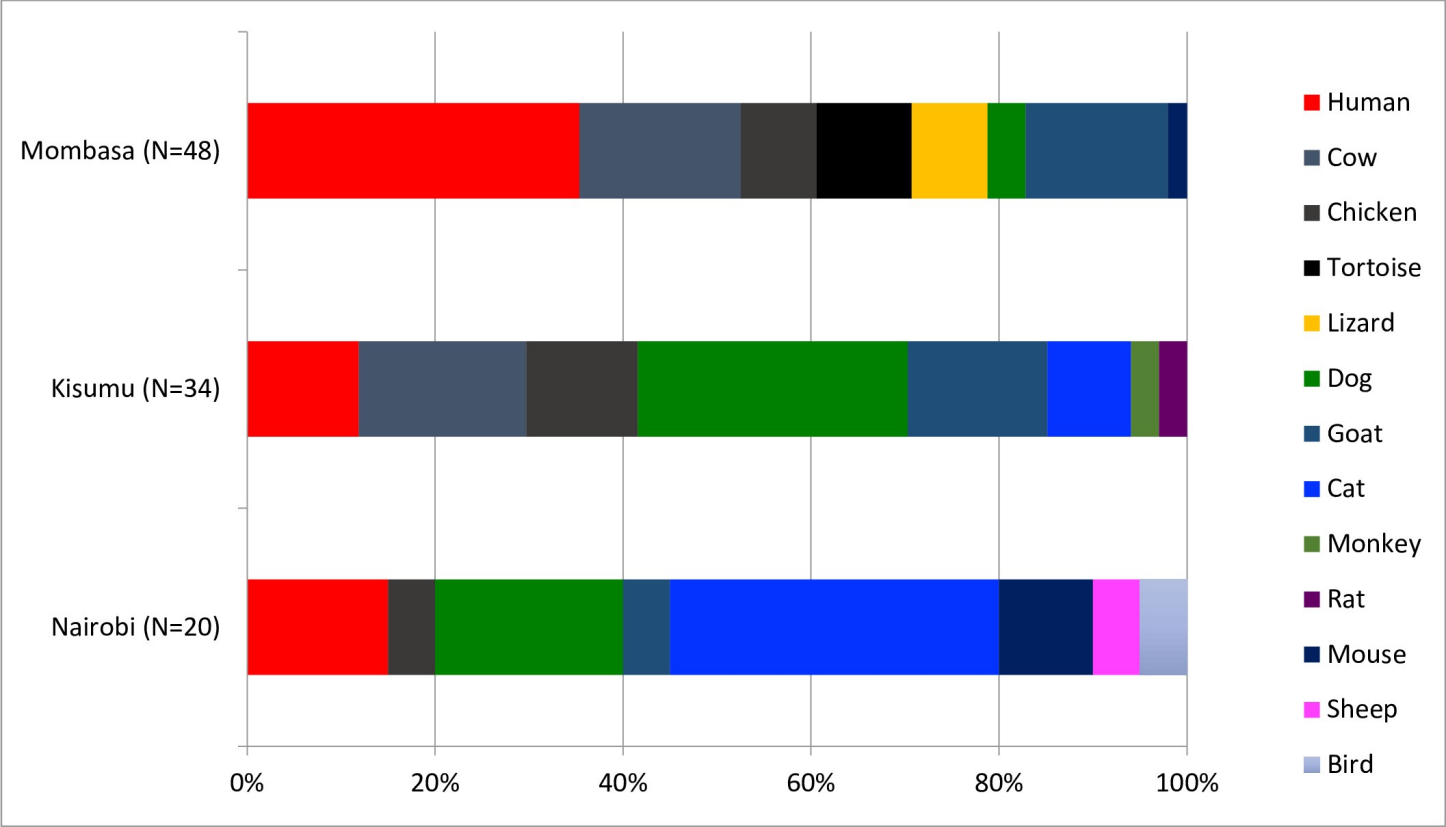
Host blood meal sources for *Aedes aegypti* mosquitoes collected from Mombasa, Kisumu and Nairobi from October 2014 to June 2016.

When examining the host blood meal sources in 102 *Ae*. *aegypti* mosquitoes from Mombasa (n = 48), Kisumu (n = 34), and Nairobi (n = 20), we identified 13 different host blood meal sources (Fig 2, S1 Table). While a significant difference was observed in *Ae*. *aegypti* human feeding between Mombasa and Kisumu (χ^2^ = 4.67, df = 1, p = 0.03), the differences between Mombasa and Nairobi (χ^2^ = 1.94, df = 1, p = 0.16), and between Kisumu and Nairobi (χ^2^ = 0.0001, df = 1, p = 1), were not significant. This translated to a human blood index of 0.4, 0.1 and 0.2 in Mombasa, Kisumu and Nairobi, respectively.

### *COI* gene variation in dengue-2 virus susceptible and non-susceptible *Aedes aegypti* mosquitoes from three urban Kenyan sites

**Fig 3:**
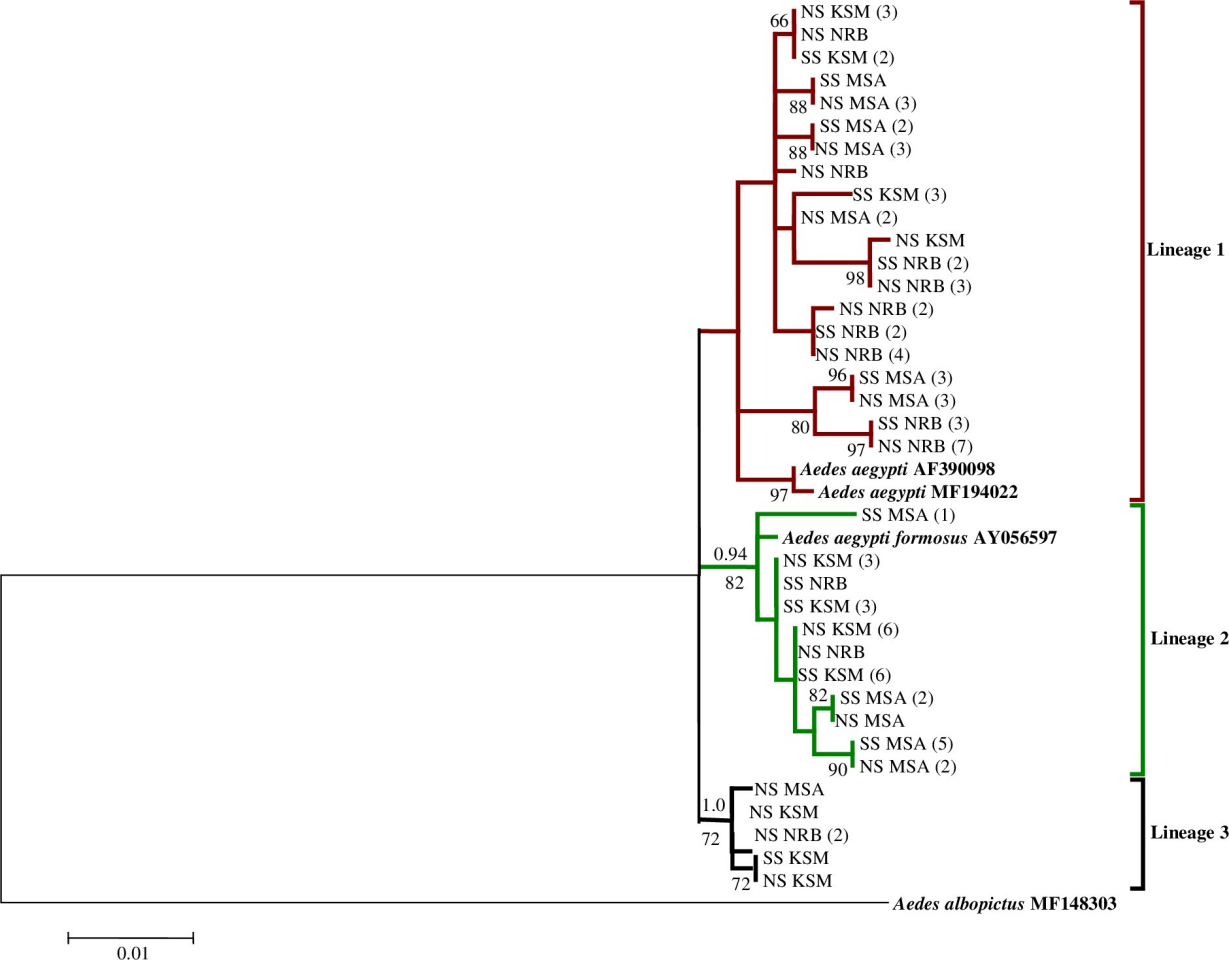
Maximum likelihood tree inferred using the (GTR+G) model of sequence evolution for *COI* barcode region (860 bp) of dengue virus susceptible (SS) and non-susceptible (NS) *Aedes aegypti* mosquitoes from Mombasa (MSA), Kisumu (KSM), and Nairobi (NRB), Kenya. The number of individuals sharing a haplotype is indicated in parentheses. Bayesian posterior probabilities ≥0.90 and bootstrap support values from 5,000 replications ≥65 are indicated above and below the three major lineages, respectively, with terminal nodes reflecting bootstrap support values alone. *Aedes albopictus* was included for outgroup purposes.

Based on phylogenetic analysis of *Ae*. *aegypti* samples that were both susceptible and non-susceptible to DENV-2 from all three cities, we identified three *Ae*. *aegypti* lineages; lineage 1 within which the domestic form (*Ae*. *aegypti aegypti -*Genbank Accession No. AF390098 and MF194022) clustered, lineage 2 containing the forest form (*Ae*. *aegypti formosus—*Genbank Accession No. AY056597), and lineage 3 which clusters within a well-supported *Ae*. *aegypti* clade (Fig 3). DENV-2 susceptible and non-susceptible *Ae*. *aegypti* were fairly represented in all three lineages (Fig 3).

## Discussion

In Kenya, during the past decade, urban dengue outbreaks remain limited to Mombasa, but not Kisumu and Nairobi [18–20]. Besides urbanization being a risk factor for the emergence of dengue, our study showed that *Ae*. *aegypti* density, feeding pattern, and prevailing environmental temperatures were important contributing factors that can differentially drive the emergence of dengue. For dengue to emerge in an area, the various risk factors must align, creating a connecting interface between the virus, the arthropod vectors, and the susceptible human population.

Differences in vector competence would be the expected explanation for the outbreaks in Mombasa, but not Kisumu and Nairobi. However, we found that populations of *Ae*. *aegypti* from all three cities had a similar vector competence for DENV-2. This suggested that differences in vector competence between the various mosquito populations does not appear to be the explanation for the differences in outbreaks.

Overall, the DENV-2 transmission rates were generally low in all three populations. This may be explained by the fact that we used the capillary tube method to estimate DENV-2 transmission. Methods collecting mosquito saliva may underestimate virus transmission [30]. Similarly, exposing mosquitoes to virus *via* a membrane feeder tends to produce a lower infection rate than feeding them on a viremic host [31,32]. However, there are currently no suitable animal models to estimate DENV transmission [33]. Because the methods of virus exposure and transmission determination were the same for all three mosquito populations, our transmission rate estimates would not be significantly affected should transmission rates increase under field conditions. As a limitation, our study only focused on DENV-2, one of the most prevalent serotype, and further studies on the other DENV serotypes (DENV-1 and -3) circulating in Kenya [18,34,35] are needed.

Similarly, environment temperature is a critical factor for the ability of *Ae*. *aegypti* to transmit DENV, with transmission rates significantly reduced at lower temperatures [17,36]. In addition, lower temperatures are known to reduce vector feeding/biting frequency [14] and can significantly reduce vector density/human-vector contact, consequently lowering the risk of DENV transmission by *Ae*. *aegypti* mosquitoes in an area [37]. This may explain the lack of dengue outbreaks in Nairobi. However, the temperatures in Mombasa and Kisumu, 27/31°C, and 28/30°C, respectively, are nearly identical. Therefore, temperature cannot explain the lack of dengue outbreaks in Kisumu.

Another possible explanation for the lack of dengue in Kisumu and Nairobi could be the density of *Ae*. *aegypti* populations. Although the *Ae*. *aegypti* density in Nairobi, was about half of that observed in Kisumu and Mombasa, and might also be one of the reasons for the lack of dengue in Nairobi, the density of *Ae*. *aegypti* was similar in Mombasa and Kisumu, so *Ae*. *aegypti* density would not explain the lack of outbreaks in Kisumu.

The absence of epidemics in Kisumu, but their presence in Mombasa, must therefore be linked to factors other than vector competence, temperature, and *Ae*. *aegypti* density. Differences in feeding behavior of the *Ae*. *aegypti* from the three locations, as determined from blood meal analysis, showed that the *Ae*. *aegypti* population from Mombasa was more anthropophilic than the population from Kisumu (Fig 2). The higher anthropophily observed in Mombasa compared to Kisumu is consistent with the observed dengue epidemics reported in Mombasa and the coastal area of Kenya at large [18–20]. Higher human blood feeding has also been reported in dengue endemic areas connoting the importance of *Ae*. *aegypti* feeding behavior in the emergence of dengue [38,39]. However, it is worth noting that the observed proportion of *Ae*. *aegypti* feeding on humans (35%) in the dengue endemic area of Mombasa was far less than the proportion recorded in other dengue endemic areas (Thailand and Puerto Rico) where *Ae*. *aegypti* feeding occurs almost exclusively on humans (80–100%) [38–40].

Feeding preference in *Ae*. *aegypti* mosquitoes has an underlying genetic basis [41,42], with the *Ae*. *aegypti aegypti* subspecies reportedly being more anthropophilic, whereas the sister taxon, *Ae*. *aegypti formosus*, is more zoophilic [42]. Thus, the low human blood feeding rates observed for the *Ae*. *aegypti* population in Kisumu may be indicative of a more zoophilic vector population composition, possibly explaining why the city is less affected by dengue.

We further observed that the *Ae*. *aegypti* population in Mombasa was not significantly more anthropophilic than that from Nairobi, suggesting that lower *Ae*. *aegypti* density and lower temperatures in Nairobi (Table 1 and Table 2), rather than mosquito feeding pattern, explained the absence of dengue from this city. To fully understand the *Ae*. *aegypti* feeding pattern, additional studies incorporating larger sample sizes are required.

Although *Ae*. *aegypti* has been reported to feed less on bovine [38], we observed about 17% feeding on cattle in both Mombasa and Kisumu (Fig 2). This can potentially be exploited in dengue, and possibly chikungunya, control by diverting *Ae*. *aegypti* feeding away from humans to insecticide-treated cows, as has been suggested for *Anopheles* mosquitoes in malaria control [43]. As a limitation, data on the density of the different host types in the study areas were not available and should be considered in future studies in order to obtain better estimates of feeding preference.

Phylogenetic analysis of DENV-2 susceptible and non-susceptible *Ae*. *aegypti* mosquitoes suggested that DENV-2 susceptibility did not vary on the basis of the *Ae*. *aegypti* subspecies/lineages present in these cities, as both the susceptible and non-susceptible *Ae aegypti* were fairly represented in all three mitochondrial lineages (Fig 3). As a limitation, the susceptible mosquitoes corresponded to mosquitoes that were shown to be infected with DENV. However, not all infected mosquitoes eventually disseminate virus, and even fewer successfully transmit virus by bite. Thus, further studies investigating the genetic basis of vector competence should consider mosquito populations with at least a disseminated virus infection.

In conclusion, our study indicated that the current concentration of dengue in coastal Mombasa, and absence of outbreaks from Kisumu, appeared to be due to differences in *Ae*. *aegypti* feeding patterns rather than differences in vector competence or environmental temperature. However, lower vector density and environmental temperature appeared to be contributory factors to the current absence of dengue outbreaks in Nairobi. Risk factors such as mosquito density, environmental temperature, vector competence, host feeding pattern, and vector genetics, when interpreted individually, may not sufficiently inform risk of transmission of DENV and must therefore be evaluated collectively. Although the risk of DENV transmission is high in Mombasa, and low in Kisumu and Nairobi, continued monitoring of DENV transmission risk and vector surveillance, as well as monitoring of contributing behavioral and environmental factors, are needed to improve early warning and pre-emptive action.

## Supporting information

S1 Protocol *Aedes aegypti* blood meal analysis. (DOCX)

S2 Protocol Assement of infection, dissemination and transmission of dengue-2 virus by *Aedes aegypti*. (DOCX)

S1 Table Vertebrate host of *Aedes aegypti* mosquitoes collected from Mombasa, Kisumu and Nairobi (2014–2016). (DOCX)

S2 Table Infection, dissemination and transmission rates of *Aedes aegypti* mosquitoes from Mombasa, Kisumu and Nairobi days post exposure to dengue-2 virus at different temperatures. (DOCX)
