# Supplementary material for: Entomological assessment of dengue virus transmission risk in three urban areas of Kenya
Source: PLoS Negl Trop Dis. 2019 Aug 23;13(8):e0007686. doi: 10.1371/journal.pntd.0007686 (PMC6728053; doi:10.1371/journal.pntd.0007686)
Supplement: S1 Protocol — (DOCX) [file pntd.0007686.s001.docx]

Genomic amplification reactions were performed in a final reaction volume of 10 µl, containing 5.0 µl of 10x Mytag HS mix, 2 µl of water, 0.5 µl (0.5 µM) of each of the forward and reverse primers (12S3F 5’-GGGATTAGATACCCCACTATGC-3’ and 12S5R 5’-TGCTTACCATGTTACGACTT-3’), and 2 µl (1-10 ng) of the genomic DNA template. The cycling conditions were 95°C for 3 mins, followed by 95°C for 20 secs, 56°C for 30 sec, 72°C for 30 sec for 35 cycles, and a final extension at 72°C for 5 mins.
