# Supplementary material for: Entomological assessment of dengue virus transmission risk in three urban areas of Kenya
Source: PLoS Negl Trop Dis. 2019 Aug 23;13(8):e0007686. doi: 10.1371/journal.pntd.0007686 (PMC6728053; doi:10.1371/journal.pntd.0007686)
Supplement: S2 Protocol — (DOCX) [file pntd.0007686.s002.docx]

For the different cities and selected temperatures, individual mosquitoes were assayed for virus infection, dissemination, and transmission (by the capillary tube method). Briefly, in batches of 10, mosquitoes were placed in a plastic cup covered with a fine netting (secured in place with a rubber band) and cold anesthetized by placing the cup at -20^o^C for 40 seconds. To prevent the mosquitoes from flying when they regained consciousness, the legs and wings of each individual mosquito were removed. The legs were placed in a microcentrifuge tube containing 500 µl of homogenization media (MEM, supplemented with 15% FBS) and frozen at -80^o^C, until assayed for virus by cell culture. The wings were discarded. The mosquito was then placed on a sticky tape and the proboscis inserted into a capillary tube containing 15-20 µl of homogenization media. The mosquito was left to salivate for 30 minutes, after which the contents of the capillary tube were eluted into a microcentrifuge tube containing 150 µl of homogenization media. This was frozen immediately at -80^o^C, until assayed for virus by cell culture. The body sample was placed in a separate microcentrifuge tube containing 500 µl of homogenization media. A copper bead (BB-caliber airgun shot) was placed inside the tube, and each body sample was homogenized using a Minibeadbeater (BioSpec Products Inc, Bartlesville, OK 74005 USA). The samples were centrifuged (Eppendorf centrifuge 5417R) at 4°C for 10 mins at a speed of 12,000 rpm to clarify the solution, after which 50 µl of the sample (one sample per well) were inoculated onto freshly grown Vero E6 cells in a 24-well cell culture plate. The cells were incubated for 1 hour to allow for virus adsorption, after which the infected cells were maintained with maintenance media. The plates were incubated at 37°C (5% CO_2_) and the cells in each well were observed daily for CPE for a period of 12 days. Wells showing CPE were recorded as potentially positive for DENV-2. The legs of mosquitoes corresponding to DENV-2 positive bodies were homogenized and tested for the virus as described above. Similarly, the saliva samples of mosquitoes with positive legs were tested as above. The supernatant of all wells showing CPE were harvested, frozen (-80^o^C) and retested to confirm virus growth. In addition, approximately 25% of the negative samples were randomly selected for a blind passage by CPE assay. Recovery of virus from the body, legs, and saliva suspension of each mosquito demonstrated infection, dissemination, and transmission of DENV-2, respectively.

Serial 10-fold dilution of the pre- and post-feeding blood/virus mixtures were prepared for virus quantification by plaque assay [25]. Briefly, 100 µl of each dilution was inoculated onto freshly grown Vero cells (confluent monolayer) in a 12-well cell culture plate. Plates were incubated for 1 hour to allow for virus adsorption, after which cells were maintained by adding 2 ml of 2.5% methylcellulose (mixed with 2X MEM), per well. After 9 days of incubation at 37°C (5% CO_2_), the media was gently poured off from each well and the cells were fixed with 10% formalin for 2 hrs. The cells were stained with 0.5% crystal violet overnight and the plaques were observed and counted using a light box.
