# Supplementary material for: Entomological assessment of dengue virus transmission risk in three urban areas of Kenya
Source: PLoS Negl Trop Dis. 2019 Aug 23;13(8):e0007686. doi: 10.1371/journal.pntd.0007686 (PMC6728053; doi:10.1371/journal.pntd.0007686)
Supplement: S1 Table — (DOCX) [file pntd.0007686.s003.docx]

|  | **Percentage (Number)** | |  |
| --- | --- | --- | --- |
| **Host** | **Nairobi** | **Kisumu** | **Mombasa** |
| Human | 15 (3) | 12 (4) | 35 (17) |
| Cow | 0 | 18 (6) | 17 (8) |
| Goat | 5 (1) | 15 (5) | 15 (7) |
| Tortoise | 0 | 0 | 10 (5) |
| Chicken | 5 (1) | 12 (4) | 8 (4) |
| Lizard | 0 | 0 | 8 (4) |
| Dog | 20 (4) | 29 (10) | 4 (2) |
| Mouse | 10 (2) | 0 | 2 (1) |
| Cat | 35 (7) | 9 (3) | 0 |
| Monkey | 0 | 3 (1) | 0 |
| Rat | 0 | 3 (1) | 0 |
| Sheep | 5 (1) | 0 | 0 |
| Bird | 5 (1) | 0 | 0 |
| **Total** | **100 (N=20)** | **100 (N=34)** | **100 (N=48)** |
