# Supplementary material for: Entomological assessment of dengue virus transmission risk in three urban areas of Kenya
Source: PLoS Negl Trop Dis. 2019 Aug 23;13(8):e0007686. doi: 10.1371/journal.pntd.0007686 (PMC6728053; doi:10.1371/journal.pntd.0007686)
Supplement: S2 Table — (DOCX) [file pntd.0007686.s004.docx]

|  |  | Rate (No. positive/No. tested) | | | | | | | | |
| --- | --- | --- | --- | --- | --- | --- | --- | --- | --- | --- |
| Days of incubation |  | 22°C | | | 28°C | | | 31°C | | |
|  |  | Mombasa | Kisumu | Nairobi | Mombasa | Kisumu | Nairobi | Mombasa | Kisumu | Nairobi |
| 7 | Infection | 8.7 (2/23) | 33.3 (4/12) | 3.8 (1/26) | 50 (9/18)* | 27.6 (8/29) | 25.0 (5/20) | 29.2 (7/24) | 52.2 (12/23) | 22.7 (5/22) |
|  | Dissemination | 0.0 (0/23) | 0.0 (0/12) | 0.0 (0/26) | 11.1 (2/18) | 0.0 (0/29) | 5.0 (1/20) | 0.0 (0/24) | 13.0 (3/23) | 13.6 (3/22) |
|  | Dissem/Infected | 0.0 (0/2) | 0.0 (0/4) | 0.0 (0/1) | 22.2 (2/9)^†^ | 0.0 (0/8) | 20.0 (1/5) | 0.0 (0/7) | 25.0 (3/12) ^†^ | 60.0 (3/5) ^†^ |
|  | Transmission | 0.0 (0/23) | 0.0 (0/12) | 0.0 (0/26) | 0 (0/15) | 0.0 (0/29) | 0.0 (0/20) | 0.0 (0/24) | 4.8 (1/21) | 0.0 (0/20) |
|  | Trans/Infected | 0.0 (0/2) | 0.0 (0/4) | 0.0 (0/1) | 0 (0/6) | 0.0 (0/8) | 0.0 (0/5) | 0.0 (0/7) | 10.0 (1/10) | 0.0 (0/3) |
|  | Trans/Dissem | 0.0 | 0.0 | 0.0 | 0 (0/2) | 0.0 | 0.0 (0/1) | 0.0 | 100 (1/1) | 0.0 (0/1) |
|  |  |  |  |  |  |  |  |  |  |  |
| 14 | Infection | 40.9 (9/22) | 38.9 (7/18) | 60.0 (3/5) | 28.6 (4/14) | 23.1 (6/26) | 11.1 (2/18) | 30.4 (7/23) | 57.1 (12/21) | 35.0 (7/20) |
|  | Dissemination | 9.1 (2/22) | 0.0 (0/18) | 0.0 (0/5) | 7.1 (1/14) | 11.5 (3/26) | 5.6 (1/18) | 21.7 (5/23) | 19.0 (4/21) | 30.0 (6/20) |
|  | Dissem/Infected | 22.2 (2/9) | 0.0 (0/7) | 0.0 (0/3) | 25.0 (1/4) | 50.0 (3/6) | 50.0 (1/2) | 71.5 (5/7) | 33.3 (4/12) | 85.7 (6/7) |
|  | Transmission | 4.5 (1/22) | 0.0 (0/18) | 0.0 (0/5) | 7.1 (1/14) | 0.0 (0/26) | 0.0 (0/18) | 8.7 (2/23) | 4.8 (1/21) | 15.0 (3/20) |
|  | Trans/Infected | 11.1 (1/19) | 0.0 (0/7) | 0.0 (0/3) | 25.0 (1/4) | 0.0 (0/6) | 0.0 (0/2) | 28.6 (2/7) | 8.3 (1/12) | 42.9 (3/7) |
|  | Trans/Dissem | 50.0 (1/2) | 0.0 | 0.0 | 100.0 (1/1) | 0.0 (0/3) | 0.0 (0/1) | 40.0 (2/5) | 25.0 (1/4) | 50.0 (3/6) |
|  |  |  |  |  |  |  |  |  |  |  |
| 21 | Infection | 37.5 (3/8) | 43.3 (8/15) | 9.1 (1/11) | 54.5 (6/11) | 32 (9/28) | 38.9 (7/18) | 7.1 (1/14) | 21.1 (4/19) | 11.8 (2/17) |
|  | Dissemination | 0.0 (0/8) | 0.0 (0/15) | 0.0 (0/11) | 27.3 (3/11) | 32.1 (9/28) | 27.8 (5/18) | 7.1 (1/14) | 21.1 (4/19) | 11.8 (2/17) |
|  | Dissem/Infected | 0.0 (0/3) | 0.0 (0/8) | 0.0 (0/1) | 50.0 (3/6) | 100.0 (9/9) | 71.4 (5/7) | 100.1 (1/1) | 100.0 (4/4) | 100.0 (2/2) |
|  | Transmission | 0.0 (0/8) | 0.0 (0/15) | 0.0 (0/11) | 0.0 (0/11) | 3.6 (1/28) | 0.0 (0/18) | 0.0 (0/14) | 10.5 (2/19) | 0.0 (0/17) |
|  | Trans/Infected | 0.0 (0/3) | 0.0 (0/8) | 0.0 (0/1) | 0.0 (0/6) | 11.1 (1/9) | 0.0 (0/7) | 0.0 (0/1) | 50.0 (2/4) | 0.0 (0/2) |
|  | Trans/Dissem | 0.0 | 0.0 | 0.0 | 0.0 (0/3) | 11.1 (1/9) | 0.0 (0/5) | 0.0 (0/1) | 50.0 (2/4) | 0.0 (0/2) |

Dissem = Dissemination.
Trans = Transmission.

* Includes one mosquito not tested for dissemination and transmission.

^†^ Includes two mosquitoes not tested for transmission.
